# Snakebite drug discovery: high-throughput screening to identify novel snake venom metalloproteinase toxin inhibitors

**DOI:** 10.3389/fphar.2023.1328950

**Published:** 2024-01-11

**Authors:** Rachel H. Clare, Charlotte A. Dawson, Adam Westhorpe, Laura-Oana Albulescu, Christopher M. Woodley, Nada Mosallam, Daniel J. W. Chong, Jeroen Kool, Neil G. Berry, Paul M. O’Neill, Nicholas R. Casewell

**Affiliations:** ^1^ Department of Tropical Disease Biology, Centre for Snakebite Research and Interventions, Liverpool School of Tropical Medicine, Liverpool, United Kingdom; ^2^ Department of Tropical Disease Biology, Centre for Drugs and Diagnostics, Liverpool School of Tropical Medicine, Liverpool, United Kingdom; ^3^ Department of Chemistry, University of Liverpool, Liverpool, United Kingdom; ^4^ Division of BioAnalytical Chemistry, Department of Chemistry and Pharmaceutical Sciences, Faculty of Science, Amsterdam Institute of Molecular and Life Sciences, Vrije Universiteit Amsterdam, Amsterdam, Netherlands

**Keywords:** venom, toxin, snakebite envenoming, SVMP, small molecule drugs, neglected tropical diseases

## Abstract

Snakebite envenoming results in ∼100,000 deaths per year, with close to four times as many victims left with life-long sequelae. Current antivenom therapies have several limitations including high cost, variable cross-snake species efficacy and a requirement for intravenous administration in a clinical setting. Next-generation snakebite therapies are being widely investigated with the aim to improve cost, efficacy, and safety. In recent years several small molecule drugs have shown considerable promise for snakebite indication, with oral bioavailability particularly promising for community delivery rapidly after a snakebite. However, only two such drugs have entered clinical development for snakebite. To offset the risk of attrition during clinical trials and to better explore the chemical space for small molecule venom toxin inhibitors, here we describe the first high throughput drug screen against snake venom metalloproteinases (SVMPs)—a pathogenic toxin family responsible for causing haemorrhage and coagulopathy. Following validation of a 384-well fluorescent enzymatic assay, we screened a repurposed drug library of 3,547 compounds against five geographically distinct and toxin variable snake venoms. Our drug screen resulted in the identification of 14 compounds with pan-species inhibitory activity. Following secondary potency testing, four SVMP inhibitors were identified with nanomolar EC_50_s comparable to the previously identified matrix metalloproteinase inhibitor marimastat and superior to the metal chelator dimercaprol, doubling the current global portfolio of SVMP inhibitors. Following analysis of their chemical structure and ADME properties, two hit-to-lead compounds were identified. These clear starting points for the initiation of medicinal chemistry campaigns provide the basis for the first ever designer snakebite specific small molecules.

## 1 Introduction

Snakebite envenoming is responsible for perhaps as many as 138,000 deaths annually, while the extensive morbidity burden of sequalae is estimated to represent around 6 million disability adjusted life years (DALYs) (Gutiérrez et al., 2017). Snakebite predominately affects rural populations of the tropics, and consequently the World Health Organization (WHO) recently classified snakebite envenoming as a priority neglected tropical disease (NTD) (Chippaux, 2017). Snakebite can result in catastrophic healthcare expenditure, as well as subsequent loss of livelihood, due to the loss or incapacitation of the primary earner in a household (Herzel et al., 2018; Harrison et al., 2019). In addition, the psychological impact of surviving a snakebite and related socio-economic implications (e.g., social stigma, ostracization and loss of time in education) can cause substantial long-term impacts on individuals and their families (Williams et al., 2011a; Harrison et al., 2019; Kasturiratne et al., 2021; Oliveira et al., 2022).

One of the major challenges that snakebite victims face is restricted access to effective treatment, since current antivenom therapies are often unavailable in the remote rural and indigenous communities that are most vulnerable to snakebite (Gutiérrez et al., 2017; Longbottom et al., 2018; Harrison et al., 2019; Beck et al., 2022). This challenge with antivenom availability is largely attributed to the need for cold chain storage and high costs associated with provision (Williams et al., 2011b). However, despite antivenom currently being the only approved therapeutic for the treatment of snakebite, these animal-derived polyclonal antibody-based therapies have several other limitations associated with them. Antivenom can only be given in a healthcare setting due to the high risk of adverse reactions and the need for intravenous administration (Williams et al., 2011a; de Silva et al., 2016) and their mode of production, i.e., raising animal polyclonal antibodies against a specific venom or venoms, results in products that are restricted in terms of the snake species they are effective against (Harrison et al., 2017; Laxme et al., 2019; Ainsworth et al., 2020).

Snake venoms are a complex mix of several protein families, peptides, and small molecules, which have evolved over millions of years to facilitate prey capture (Tasoulis and Isbister, 2017; Tasoulis et al., 2021). Snake venoms often display compositional variation, and while this is most commonly observed between snake species, there are also several examples of extensive intraspecific and ontogenetic venom variation reported in the literature (Chippaux et al., 1991; Currier et al., 2010; Casewell et al., 2020; 2014; Amazonas et al., 2018; Laxme et al., 2019; Oliveira et al., 2019). Venom variation results in variation in toxin functionality, with associated human snakebite pathologies being equally diverse; the most prominent being neurotoxicity (neuromuscular paralysis), cytotoxicity (local swelling, blistering and tissue necrosis) and haemotoxicity (haemorrhage and coagulopathy) (Warrell, 2010; Gutiérrez et al., 2017; Slagboom et al., 2017; Longbottom et al., 2018). Of the many venom components found across medically important venomous snakes (i.e., families Viperidae and Elapidae), four toxin families are considered amongst the most important therapeutic targets due to their role contributing to these pathologies: three finger toxins (3FTx), snake venom serine proteases (SVSP), phospholipases A_2_ (PLA_2_) and snake venom metalloproteinases (SVMP) (Ferraz et al., 2019).

Snake venom metalloproteinases (SVMPs) are zinc-dependent endoproteolytic toxins that evolved from ADAM genes (Moura-da-Silva et al., 1996; Casewell et al., 2012). These toxins are found in the venoms of almost all medically important snakes, though they are most abundant in viperids, where they can account for up to 74% of total venom composition (average, 36%) (Tasoulis and Isbister, 2017; Dawson et al., 2021). SVMPs can be broadly divided into three classes based on their tertiary structure (P-I, P-II, and P-III), with each being progressively larger and more structurally complex due to the presence of additional domains (Fox and Serrano, 2008; 2005). Haemorrhage is the most pathologically serious complication resulting from SVMP activity and can occur both systemically and locally. The degradation of collagen and other key extracellular matrix proteins by SVMPs weakens the vascular endothelium, with the impact of haemodynamic forces on this weakened endothelium then enabling blood to escape from the vessels (Gutiérrez et al., 2016; Asega et al., 2020). In addition, several SVMPs also target different points in the coagulation cascade, particularly the common pathway components, fibrinogen, prothrombin and factor X (Kini and Koh, 2016; Asega et al., 2020). These key clotting factors are then consumed, either by their activation or degradation, ultimately leading to the depletion of fibrinogen and, in severe cases, venom induced consumption coagulopathy (Maduwage and Isbister, 2014; Kini and Koh, 2016).

Recent innovations in snakebite therapeutics have centred around the use of monoclonal antibodies or small molecule toxin inhibitors (i.e., drugs), with the latter focusing on their potential application against PLA_2_ and SVMP rich venoms (Lewin et al., 2016; Knudsen and Laustsen, 2018; Albulescu et al., 2020b; Clare et al., 2021; Hall et al., 2022; Menzies et al., 2022). So far, two groups of molecules with differing modes of action have been found to inhibit SVMP activity. The first are metal chelators that bind Zn^2+^; the leading candidate from this class is 2, 3-Dimercapto-1-propanesulfonic acid (DMPS: Unithiol), a drug licensed for the treatment of heavy metal poisoning, marketed under the name Dimaval^®^, and available in both oral and parenteral forms (Drisko, 2018). DMPS exhibits efficacy in *in vivo* models of snakebite envenoming against venoms rich in SVMP toxins (Albulescu et al., 2020a; Menzies et al., 2022), and recently completed Phase I dose optimisation studies for snakebite indication (Abouyannis et al., 2022). The second drug group are the matrix metalloproteinase inhibitors (MMPi); these compounds include a zinc-binding motif and directly interact with the active site of these enzymes (Gutiérrez et al., 2021). Marimastat has emerged as an exciting early candidate from this group; initially developed as an anti-cancer drug, marimastat passed into Phase III clinical trials, where ultimately development was halted due to lack of efficacy (Winer et al., 2018). Marimastat exhibits broad and potent inhibition of SVMP toxins *in vitro* (Xie et al., 2020; Menzies et al., 2022) and exerts promising preclinical efficacy against the local and systemic haemotoxic effects of several snake venoms in small animal models (Albulescu et al., 2020b; Hall et al., 2022; Menzies et al., 2022). Like DMPS, marimastat exhibits desirable oral bioavailability (Millar et al., 1998), which is an exciting characteristic for future snakebite therapeutics, as drugs could potentially be rapidly administered in rural communities soon after a bite. Reducing current delays to snakebite treatment is likely to substantially improve patient outcomes (Gutiérrez et al., 2017). One of the other major potential benefits of drugs like DMPS and marimastat is the opportunity to prioritise those with existing clinical data or regulatory approvals, which could result in major reductions in the time and costs associated with translating drugs to market (Ashburn and Thor, 2004).

Although both DMPS and the PLA_2_ inhibiting drug varespladib have moved into clinical development for snakebite indication (Abouyannis et al., 2022; Carter et al., 2022), the snakebite drug portfolio remains extremely limited, with attrition a major risk. There is a strong need to develop a broad portfolio of potential snakebite drugs in the early hit discovery stage to allow the selection of candidates with the highest chance of success, and to provide multiple back-up compounds should a primary hit series fail during hit-to-lead optimisation.

Consequently, in this study we performed a repurposed drug screen to identify novel SVMP toxin-inhibiting drugs. We first report on the validation of a high-throughput primary screen capable of assessing SVMP inhibition and processing ∼3,500 compounds per day. Next, we performed a drug discovery campaign by screening a commercial human pharmacopoeia library consisting of 3,547 post-Phase I drugs, with the aim of identifying hits for direct repurposing or as starting points for future medicinal chemistry campaigns. The results of our screening campaign, the first of its kind applied in the context of snakebite, yielded four novel compounds with promise for downstream development.

## 2 Materials and methods

### 2.1 Venoms

Venoms were extracted from specimens of *Echis ocellatus* (likely *Echis romani* following recent taxonomic reclassification (Trape, 2018), and awaiting molecular confirmation) (Nigeria), *Crotalus atrox* (United States), and *Bitis arietans* (Nigeria) maintained within the herpetarium at the Centre for Snakebite Research and Interventions (CSRI) at the Liverpool School of Tropical Medicine (LSTM). Crude venoms were pooled by species and lyophilised for long term storage at 2°C–8°C. Venoms from *Calloselasma rhodostoma* (Thailand) and *Bothrops jararaca* (Brazil) were sourced from the historical collection of lyophilised venoms at LSTM. Venoms were reconstituted to 10 mg/mL in sterile Phosphate Buffered Saline (PBS) (Cat no: 20012027, ThermoFisher) prior to use.

### 2.2 Drug stocks

Several matrix metalloproteinase inhibitors were commercially sourced, namely, marimastat ((2*S*,3*R*)-*N*4-[(1*S*)-2,2-Dimethyl-1-[(methylamino)carbonyl]propyl]-*N*1,2-dihydroxy-3-(2-methylpropyl)butanediamide, ≥99%, Cat no: 2631, Tocris Bioscience), prinomastat hydrochloride (AG3340 hydrochloride, ≥98%, Cat no: HY-12170A, MedChemExpress), tanomastat (4′-chloro-γ-oxo-αS-[(henylthiol)methyl]-[1,1′-biphenyl]-4-butanoic acid, ≥98%, Cat no: 19258, Cayman chemicals), batimastat ((2R,3S)-N4-Hydroxy-N1-[(1S)-2-(methylamino)-2-oxo-1-(phenylmethyl)ethyl]-2-(2-methylpropyl)-3-[(2-thienylthio)methyl]butanediamide, ≥98%, Cat no: SML0041, Sigma) and doxycycline (6-Desoxy-5-hydroxytetracycline hydrochloride hemihydrate hemiethanolate, ≥93.5%, Cat no: D9891, Sigma). The metal chelators dimercaprol (2,3-dimercapto-1-propanol, ≥98% iodometric, Cat no: 64046, Sigma) and DMPS (2,3-dimercapto-1-propane-sulfonic acid sodium salt monohydrate, 95%, Cat no: H56578, Alfa Aesar) were also commercially sourced. Small molecules with alternative toxin targets were tested as negative drug controls; varespladib (2-[[3-(2-Amino-2-oxoacetyl)-2-ethyl-1-(phenylmethyl)-1H-indol-4-yl]oxy]-acetic acid, ≥98%, Cat no: SML1100, Sigma) and nafamostat mesylate ((6-Carbamimidoylnaphthalen-2-yl)4-(diaminomethylideneamino)benzoate methanesulfonic acid, > 97%, Cat no: ab141432, Abcam).

For the primary high-throughput screen (HTS) a bespoke repurposed drug library (a combination of the HY-L035 and HY-L026 libraries with overlaps removed) was purchased from MedChemExpress. The library was curated by MedChemExpress to contain a diverse chemical panel of 3,547 post-Phase I and approved drugs that have completed preclinical and clinical studies for a wide range of diseases, as well as having well-characterised bioactivity, safety, and bioavailability properties, making them suitable for drug repurposing (Supplementary Datasheet).

### 2.3 Drug preparations

Drugs were either supplied at 10 mM or reconstituted in dimethyl sulfoxide (DMSO) (≥99.7%, Cat no: D2650, Sigma) to the same concentration and stored at −20°C. Daughter plates were created with 40 µL per well of 1 mM stocks on V-bottomed 384-well plates (Cat no: 781280, Greiner) to allow the creation of assay-ready plates using a VIAFLO 384 electronic pipette (Integra), excluding columns 1 and 24. Daughter plates and assay-ready plates were stored at −20°C, with the latter used within a month of creation. For SVMP assay-ready plates, 0.91 µL of each drug from the respective daughter plate was plated, such that in the final assay volume of 91 μL, a final concentration of 10 µM was reached. Controls were processed in a similar manner into columns 1 and 24 of each plate; DMSO, PBS, 10 µM marimastat, and 50 nM marimastat, with each condition repeated 4 times within each control column. In secondary screening, dose response curves were created, with a top final assay concentration of 10 µM and reaching sub-nanomolar concentrations (concentration range was dependent on specific plate layout), with each concentration tested in duplicate. For DMPS and dimercaprol, dose response curves started at a concentration of 160 µM to account for reduced inhibitory potency compared to other compounds, with each concentration tested in duplicate.

### 2.4 *In vitro* drug screening—neutralisation of venom SVMP activity

The SVMP activity of crude venom in the presence of inhibitors or vehicle control (DMSO) was measured using a quenched fluorogenic substrate (substrate for MMPs, ADAMs and Cathepsins, Cat no: ES010, Bio-Techne). The substrate was diluted in reaction buffer (150 mM NaCl, 50 mM Tris-HCl pH 7.5) and used at a final assay concentration of 7.5 µM (supplied as a 6.2 mM stock). Reactions consisted of 1 µg of venom in 15 µL PBS co-incubated with 0.91 µL of inhibitor at a concentration of 1 mM. The 384-well plate (Cat no: 781101, Greiner) was briefly centrifuged in a Platefuge (Benchmark Scientific) and incubated at 37°C for 25 min. Following incubation, the plate was acclimatised to room temperature for 5 min, before the addition of freshly prepared fluorogenic substrate (75 µL of 9.2 µM). The plates were read on a CLARIOstar microplate reader (BMG Labtech), at an excitation wavelength of 320–10 nm and emission wavelength of 420–10 nm with 10 flashes per well at 25°C, at a pre-defined end read time. The end-read times were selected independently for each venom, as the plateau point for the venom + vehicle control (maximum fluorescence) identified in an initial 1-h kinetic read.

The SVMP activity for the end-read data was calculated for each test condition as a percentage of the mean of the venom + vehicle wells (100% activity), with a baseline of the marimastat 10 µM controls representing 0% activity (Supplementary Equation S1). For the dose response screens, absolute half maximal effective concentration (EC_50_) values were calculated from the percentage inhibition values by fitting a nonlinear regression curve for the normalised response (variable slope) for each compound using GraphPad Prism 9.0 (GraphPad Software, San Diego, United States).

In addition, these controls were used to calculate the Z prime (Z′) as a quality control measure for each screening plate (Supplementary Equation S2).

### 2.5 Chemistry prioritisation

The repurposed HTS library was screened for common Pan-Assay Interference compoundS (PAINS) substructures (Baell and Holloway, 2010) using the in-built PAINS filter in the RDKIT cheminformatics package version 2022.03.5 as implemented in Python (Landrum, 2006). Tree Manifold Approximation Projection (TMAP) visualisation was performed using the TMAP python module version 1.0.6. (Probst and Reymond, 2020). ADME properties of hit molecules and radar plots were obtained using the SwissADME web server (Daina et al., 2017). Compounds were hand-picked based on their predicted ADME properties and potential further optimisation.

## 3 Results

### 3.1 Optimisation for high-throughput capability

**FIGURE 1:**
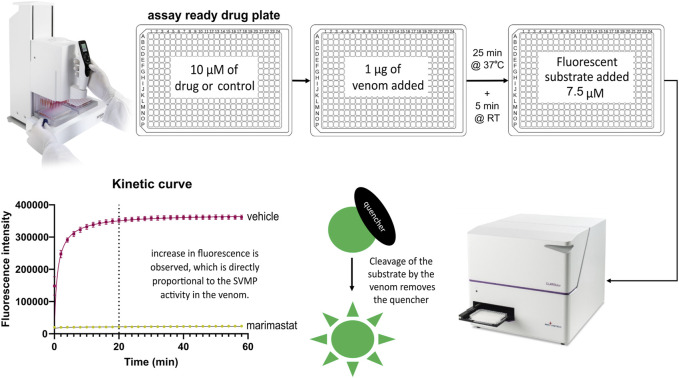
High-throughput SVMP screening workflow. This three-step screen is completed within 2 hours. Master drug plates are created with selected drugs of interest and control samples including negative vehicle controls (DMSO) and positive controls (the SVMP inhibiting MMPi marimastat). Using the VIAFLO 384 handheld electronic pipette (Integra), assay-ready drug plates are created by adding 0.91 µL of compound from a master plate. To initiate the assay, 1 µg of venom is added and the plate is incubated for 25 min at 37°C, followed by a 5 min acclimatisation to room temperature (RT). The fluorescence-quenched peptide substrate is added to all wells at a final concentration of 7.5 µM with a final assay volume of 91 µL including 10 µM of drug. Following an incubation time selected based on the plateau point previously determined by a kinetic read (e.g., 20 min for *Echis ocellatus* in this figure) the plate is read with triplicate endreads. Active drugs inhibit the ability of the venom to cleave the quencher off the fluorescent substrate, resulting in a low fluorescent read out, whereas inactive drugs will give a high fluorescence read due to uninhibited venom activity.

The SVMP assay implemented here has previously been used for quantifying venom activity and toxin inhibition and is based on the adapted use of a commercial assay designed for human MMPs (Albulescu et al., 2020b; Hou et al., 2021; Menzies et al., 2022; Nguyen et al., 2022). The fluorescence-quenched peptide substrate measures the activity of peptidases such as SVMPs which cleave the amide bond between the fluorescent group and the quencher group, resulting in an increase in fluorescence. When metalloproteinase activity is inhibited, the quencher is undisturbed, resulting in only background fluorescence. To increase the throughput of this assay, we implemented the use of robotics and associated adaptation of the method described by Albulescu et al., 2020b, including reducing the substrate concentration from 10 μM to 7.5 µM to minimise the cost of completing a high-throughput screen. The 384-well assay was semi-automated by utilising a VIAFLO 384-channel pipette which allowed single-step addition of both the venom and substrate to the assay-ready drug plates. This two-step protocol, in conjunction with assay ready drug plates, could be utilised to test a panel of venoms in a single batch run. The capacity to create assay-ready plates was provided by the VIAFLO 384-channel pipette’s ability to stamp out multiple plates from manually created master drug plates, enabling creation of either single-dose primary screening or downstream dose-response plates. The finalised high-throughput-compatible method is demonstrated in Figure 1.

**FIGURE 2:**
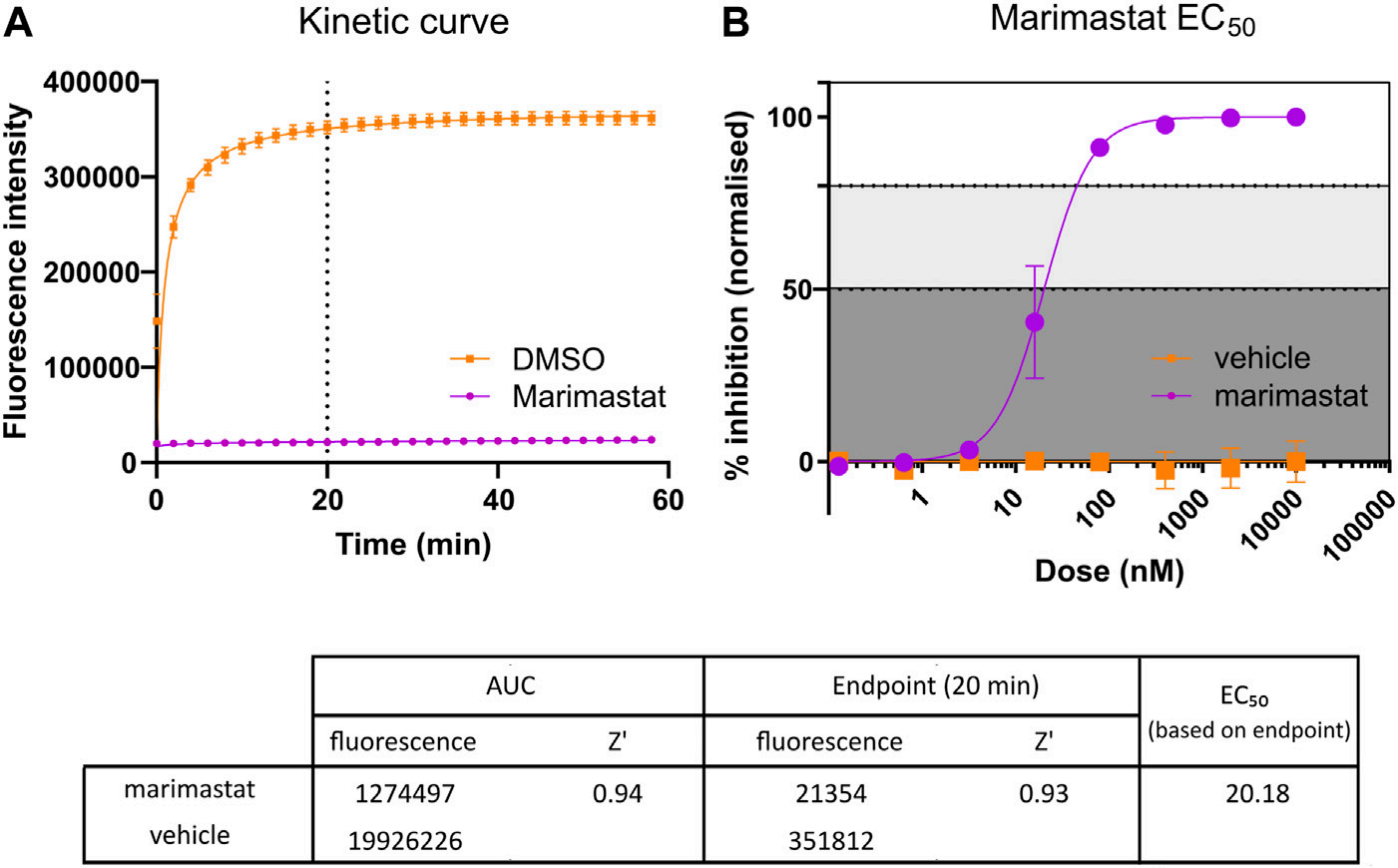
Comparison of kinetic Area Under Curve (AUC) measurement and endpoint measurement. **(A)** Kinetic curve measurement of positive (10 µM marimastat + venom) and negative (DMSO vehicle + venom) controls. Following the protocol outlined in Figure 1, kinetic measurement was taken for 60 min, and the AUC measurement calculated (*n* = 96). Dotted line indicates timepoint at which comparative endpoint reads were taken (20 min). **(B)** EC_50_ curve generated from positive and negative controls ran as a dose response plate created, starting at 10 µM and diluting 1:5 down to 128 pM. Raw data from the 20-min timepoint was normalised to a percentage inhibition based on the positive (100%) and negative (0%) controls and plotted in a dose-dependent manner (*n* = 2). The table demonstrates the raw values and Z′ values for both methods of response calculation. Z′ values were calculated by comparison of positive and negative raw values across the respective test plates. Z′ values range from 0 to 1, with values closer to 1 indicating clearly defined positive and negative controls, with little to no overlap in values between controls. The EC_50_ of marimastat was calculated through nonlinear trendline fit ([inhibitor] vs. normalized response—Variable slope) using Graphpad Prism version 9.

Through the adaptation to using end-point measurements rather than the original 1-h kinetic measurement, the time required to read a single plate was reduced to less than 100 s. The endpoint was selected as the plateau point of substrate cleavage, measured in triplicate. This endpoint was dependent on the SVMP activity profile of the individual venoms selected; for example, the endpoint for *E. ocellatus* venom was selected as 20 min post substrate addition (drug vehicle control, DMSO in Figure 2A). This provided the capacity to stagger multiple plate batches when working with large drug libraries, thus increasing the throughput capacity of the assay from a maximum of seven plates/day for a kinetic read to a maximum of 21 plates/day with this endpoint read, whilst maintaining a strong assay window.

The aim of this screening campaign was to identify novel molecules with activity at <10 µM in the primary screen and, ideally, exhibiting low nanomolar activity EC_50_ in secondary screening comparable to marimastat’s EC_50_ demonstrated in Figure 2B (Clare et al., 2019). Based on this, positive (10 µM marimastat) and negative (vehicle) controls were included on all screening plates as a quality control assessment. For *E. ocellatus* there was a 16-fold assay window between the fluorescence of the controls at the selected 20-min endpoint (351,812 and 21,354 for vehicle control and marimastat, respectively) (Figure 2A). Furthermore, to align with industry practice for drug discovery campaigns, the controls were used to calculate Z′ for each plate to monitor the assay window and variation in the data as an additional quality control step (Zhang et al., 1999). The Z′ is indicative of how discrete each control is; the closer the Z′ value is to 1, the less likely there is to be overlap between the positive and negative control values. Therefore, the Z′ of 0.93 emphasises the robust nature of this readout due to a combination of large assay window and minor variation in the data from 96 samples per control (Figure 2A).

### 3.2 Validation of the high throughput screen

To ensure that the optimised methodology was robust for HTS, we assessed intra- and inter-plate signal uniformity across triplicate 384-well plates containing either the negative control (vehicle control; plates 1–3) or the positive control (10 µM marimastat; plates 4–6). As seen in Supplementary Figure S1A, there was signal uniformity both horizontally and vertically across each plate (coefficient of variation [CV%] between 2.42 and 3.93), though there was a degree of inter-plate variance in raw signal values between repeated experiments, as best evidenced by reduced signal in plate 1 compared to plates 2 and 3 (mean 270,148 vs. 360,521 and 391,484 fluorescence AU, respectively). However, the assay window was still greater than 11-fold between the lowest vehicle control signal (plate 1) and the highest marimastat signal (plate 5). Normalising the data to percentage of inhibition based on the positive and negative controls, with 10 µM marimastat representing 100% inhibition, corrected this variance (Supplementary Figure S1B) and allowed for inter-plate consistency across a HTS for identifying drug hits.

Following validation of positive and negative controls, we then determined whether there was any inter-well interference when control wells were interleaved; as this is a fluorescence-based assay, there is potential for a high-signal well to bleed over into low-signal wells and be erroneously detected. Consequently, full rows of wells in duplicate (i.e., rows A + B, C + D, etc.) were plated containing PBS, DMSO, or marimastat at either 10 µM or a suboptimal 50 nM concentration (Supplementary Figure S2). Minimal variance was observed between the positive (raw signal range from 275,763–409,939 across all plates) and negative (raw signal range from 18,037–27,605 across all plates) controls, confirmed by a CV% from the raw data of 3.24% or lower across all conditions from all plates, and resulting in Z′ of 0.89–0.94. Although there is variation in the 50 nM control (percentage inhibition ranges from 21.7% to 78.7%), the condition is clearly discrete from the positive and negative readings. In addition, no spill-over into the previously defined strong hit classification was detected (>80% inhibition) (Supplementary Figure S2).

**FIGURE 3:**
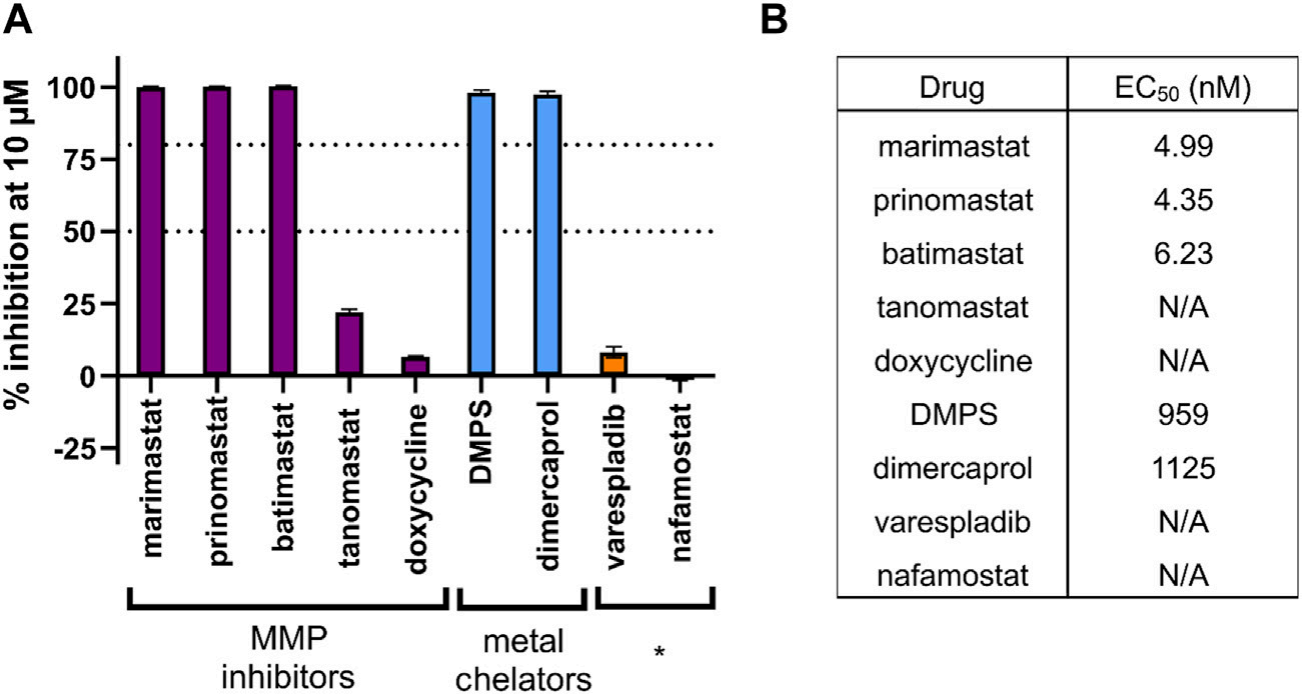
The inhibitory potency of various drugs against the SVMP activity of *E. ocellatus* venom using the validated HTS assay. **(A)** Bar graph demonstrating the percentage inhibition (when normalised to positive and negative controls) of compounds tested through the screening method. These include chemical groups broadly known to inhibit SVMPs, namely, matrix metalloproteinase inhibitors (MMPi) and metal chelators. * indicates known inhibitors of other snake venom toxin families (PLA_2_ and serine proteases) used as negative controls. Dotted lines are present at the cutoff values for mediocre (50% inhibition) and strong (80% inhibition) hit identification. **(B)** EC_50_ data calculated for each compound from the 20-min endpoint read data. Compounds with an EC_50_ displaying N/A did not cross the 50% inhibition threshold at any dose and therefore EC_50_s could not be calculated.

As a validation screen, we tested a panel of drugs known to inhibit the activity of snake venom toxins. These included metal chelators (DMPS and dimercaprol) and a broad range of MMPis (marimastat, prinomastat, batimastat, tanomastat and doxycycline). In addition, we included the drugs varespladib and nafamostat which are known to target other snake venom toxins (PLA_2_ and SVSP, respectively), but not SVMPs, as negative controls. The observed EC_50_s were as expected based on prior experience, with marimastat, prinomastat and batimastat all having strong SVMP inhibitory activity into the low nanomolar range while the metal chelators, which are known to have lower potency in this assay, displayed EC_50_s in the low micromolar range (Figure 3). As expected, the negative control drugs exhibited no activity at the top 10 µM dose tested, and therefore EC_50_ curves were not created (Figure 3).

**FIGURE 4:**
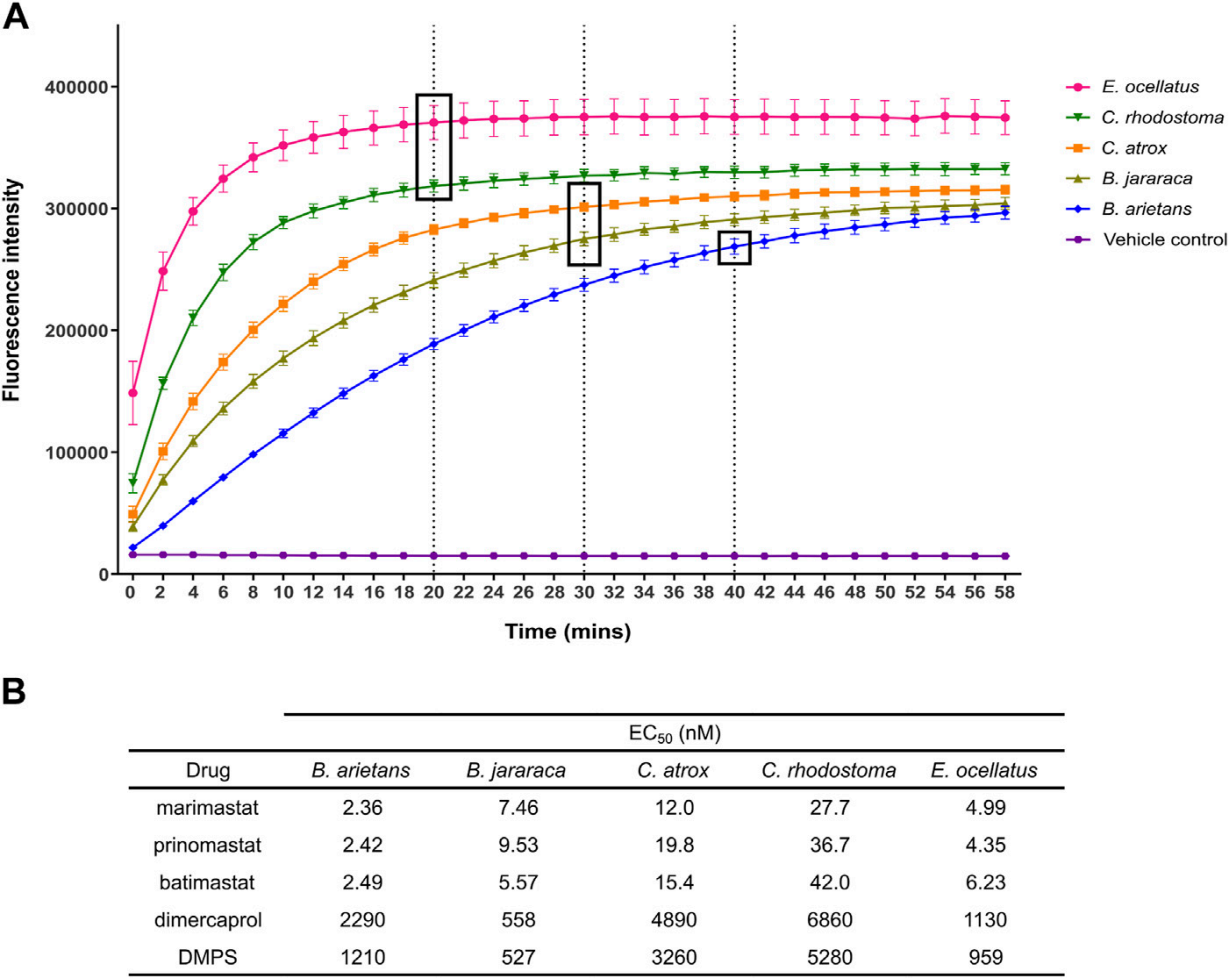
Identification of endpoint read times for each venom in the panel, and example EC_50_s for each venom from the panel of known inhibitors. **(A)** Kinetic read of venom-only activity in the SVMP assay to identify endpoint read times. Timepoint selection was based on the time at which quencher cleaving had predominantly ceased, as indicated by a plateau in fluorescence values. Venoms with greater SVMP activity generally demonstrated earlier plateau (*E. ocellatus, C. rhodostoma*) when compared to other venoms. Dotted lines and boxes indicate selected timepoint for the respective venoms. **(B)** Table showing EC_50_ values of SVMP inhibitors from the known inhibitors when screened against the panel of five viper venoms as a dilution series.

The strong, consistent Z′ results and clear signal windows from these validation experiments provided confidence to expand this approach to four other medically important viper venoms. These came from four distinct viper genera (family *Viperidae*) found on four different continents, and were selected to account for well-known variations in venom composition among snakes (Tasoulis and Isbister, 2017; Casewell et al., 2020), including variable SVMP content (Table 1). All venoms were initially measured using the original kinetic read method of the assay, and their fluorescence profile examined to determine the plateau point of their respective fluorescence signals (Figure 4A). Upon interrogation of these profiles, the following time points were selected for endpoint read data collection: *Echis ocellatus* and *Calloselasma rhodostoma,* 20 min; *Crotalus atrox* and *Bothrops jararaca*, 30 min; *Bitis arietans,* 40 min.

**TABLE 1:** Details of the venoms tested in the HTS and statistics on the resulting primary screen.

Snake species	Geographical range	SVMP % content	Z prime range	Hit rate (%)	
				Strong hits	Mediocre hits
Echis ocellatus*	West Africa	37.5% (Nigeria)	0.74–0.94	23 (0.6)	4 (0.1)
*Crotalus atrox*	South United States and Mexico	49.7% (United States)	0.76–0.94	19 (0.5)	5 (0.1)
*Calloselasma rhodostoma*	South-east Asia	41.2% (Indonesia, Malaysia)	0.78–0.96	17 (0.5)	5 (0.1)
*Bothrops jararaca*	Central South America	10.3–35.6 (Brazil)	0.71–0.88	35 (1.0)	37 (1.0)
*Bitis arietans*	Throughout sub-Saharan Africa	21.1% (Nigeria)	0.74–0.91	21 (0.6)	22 (0.6)

Geographical range is taken from the IUCN, redlist. SVMP content sourced from Casewell et al., 2014 (*E. ocellatus*), Calvete et al., 2009 (*C. atrox*), Tang et al., 2016 (*C. rhodostoma*), Gonçalves-Machado et al., 2016 (*B. jararaca*), and Dingwoke et al., 2021 (*B. arietans*). *species likely to be *E. romani* following recent taxonomic reclassification (Trape, 2018).

The same inhibitors that were used to validate the initial experiments with *E. ocellatus* venom were then applied to these additional venoms (Figure 4B). The same potency differences between the metal chelators and the MMPis were observed with the four new venoms with EC_50_s of >500 nM maintained for the metal chelators compared to <50 nM for the MMPis. This potency difference between the two drug classes is likely due to their distinct mechanism of action (Albulescu et al., 2020b; Xie et al., 2020). There were also differences between drugs of the same class; across all venoms, DMPS produced lower EC_50_ values relative to dimercaprol (DMPS = 527–5,284 nM range across all venoms, average 2,248 nM; dimercaprol = 558–6,860 nM, average 3,144 nM). Between the MMPis, minimal difference was observed between the drugs for each of the venoms tested, with all EC_50_ values lower than 50 nM against all venoms. However, for all drug classes, the EC_50_s were higher (∼2–20 fold) against *C. rhodostoma* and *C. atrox* venoms compared to those obtained for *B. arietans*, *B. jararaca* and *E. ocellatus* venom.

### 3.3 Larger-scale screening of a repurposed drug library

Following the full validation of the assay, we next embarked on a HTS of a repurposed drug library consisting of 3,547 post-Phase I compounds. Screening of the 11-plate library at 10 µM against a single venom was possible in approximately 2 hours, depending on the venom being used for screening (20-min plateau time required 2 hours, 40-min plateau time required 2 hours and 20 min). This throughput meant that the full screen of the library against all five venoms was completed within 5 days. Consistent Z′ of between 0.71–0.96 across all 11 screening plates for each venom (total of 55 × 384 well plates) indicated that the robustness of the assay was retained during the screening campaign (Table 1).

Normalised screening results were characterised into three discrete groups; non-hits (≤50% inhibition compared to control), mediocre hits (50%–80% inhibition), and strong hits (>80% inhibition). The hit rate for strong hits (>80% inhibition) ranged from 0.5% to 1.0% depending on the venom screened, with the highest rate of strong hits observed against *B. jararaca* venom (35 hits, 1.0%) (Table 1). The lowest hit rates were observed against *C. rhodostoma* and *C. atrox* venoms (17 and 19 hits respectively; both ∼0.5%). A greater disparity was observed in the number of mediocre hits recovered (>50% < 80% inhibition), with the highest number of hits observed against *B. jararaca* venom (37 hits, 1.0%), followed by *B. arietans* (22 hits, 0.6%). Far fewer mediocre hits were observed against the remaining venoms (*E. ocellatus,* 4; *C. atrox,* 5; *C. rhodostoma*, 5), each representing only 0.1% of the library. It is worth noting that this order of hit rate correlates with the rank ordering of EC_50_ potencies described earlier for known SVMP inhibitors, with *C. rhodostoma* and *C. atrox* venom seemingly being more challenging to inhibit.

**FIGURE 5:**
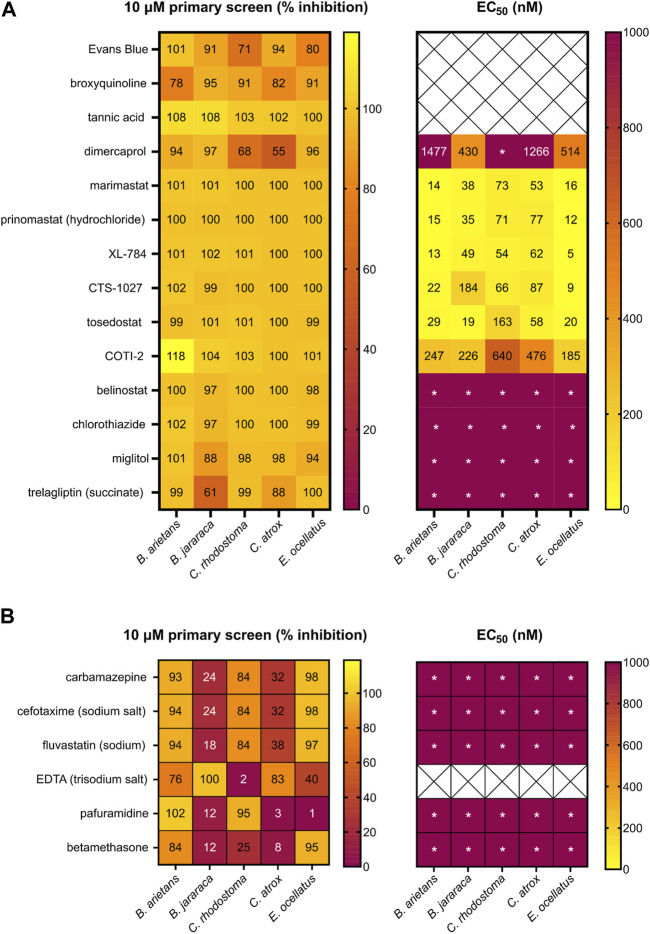
Heatmap of the hits from the primary and secondary EC_50_ screen of the repurposed compound library. Left panels present the primary screen at 10 µM and right panels present the secondary dose response screen. **(A)** Percentage inhibition of 14 compounds identified as strong hits in three or more venoms during the primary screening of the repurposed library (left) and EC_50_ data of 11 compounds progressed into the secondary screen (right). **(B)** Percentage inhibition for six strong hits in the primary screen against at least two venoms (left) and EC_50_ (right). Wells demonstrating * indicate an EC_50_ was not calculated due to low activity at the highest doses.

To prioritise hits for progression into secondary EC_50_ screening, recovered hits were assessed for their consistent activity across all five venoms (Figure 5). In total, we had 109 compounds that were either a mediocre or strong hit in at least one venom, but of these, only 10 were strong hits in all five venoms. A further four were strong hits in at least three venoms and mediocre in the additional two venoms. These 14 lead hits are presented in Figure 5A. There were a further six compounds with strong activity in two or three venoms, but lacking activity (<50% inhibition) in the additional venoms (apart from ethylenediamine tetracetic acid (EDTA), which displayed strong activity in two venoms and mediocre in a third) (Figure 5B). Of the remaining 89 compounds, inhibitory activity varied between venoms, with 32 compounds (36%) identified as strong hits against one venom (mainly *B. jararaca*) but lacking activity in the remaining four venoms. This emphasises the value in testing several venoms to maximise the chance of identifying pan-species inhibitory activities.

Of the 14 hits with pan-species activity (Figure 5A), all except three were progressed into secondary EC_50_ testing. Evans Blue and tannic acid were not considered further due to their high molecular weights coupled with known polypharmacology, while broxyquinoline was considered an intractable starting point due to previously reported toxic side effects (Strandvik and Zetterström, 1968; Baumgartner et al., 1979). The remaining 11 pan-species hits included the known and previously described SVMP inhibitors dimercaprol, marimastat and prinomastat (Rucavado et al., 2000; Ainsworth et al., 2018; Albulescu et al., 2020b; Chowdhury et al., 2021). These were progressed as additional controls in the EC_50_ screen and exhibited inhibitory potencies as anticipated (i.e., Figure 5A in comparison with Figure 4B).

The remaining eight novel hits fell into three broad inhibitory categories when tested against the five venoms in this secondary screen (Figure 5A). Three drugs (XL-784, CTS-1027 and tosedostat) had comparable EC_50_ activity (5–184 nM) to marimastat and prinomastat across all venoms. COTI-2 was slightly less active, producing EC_50_s that were approximately 10-fold lower, though these were still superior (>2 fold) to dimercaprol across all venoms (EC_50_s, 185–640 nM). The four remaining drugs (belinostat, chlorothiazide, miglitol and trelagliptin) were less active than dimercaprol, with EC_50_s greater than 1 µM against each of the venoms.

Although discovery of drug compounds with pan-species activity was the overarching goal of this project, hits with strong activity against two or more venoms were also progressed to EC_50_ testing (Figure 5B) to ensure valuable hits had not been excluded (e.g., false negatives against certain venoms). We still excluded EDTA since it has been investigated previously as a potential snakebite therapy and was found to be inferior to other metal chelators (DMPS and dimercaprol), whilst having poor therapeutic characteristics; high affinity for calcium, poor safety profile, and requirement for slow intravenous administration (Flora and Pachauri, 2010; Ainsworth et al., 2018; Albulescu et al., 2020a). Of the remaining six hits in this category (Figure 5B), when tested in the EC_50_ screen, all displayed poor activity against all venoms (EC_50_s > 1 µM), thus further validating the focus on hits with pan-species activity in the primary screen.

### 3.4 Chemical space visualisation of the hit compounds

**FIGURE 6:**
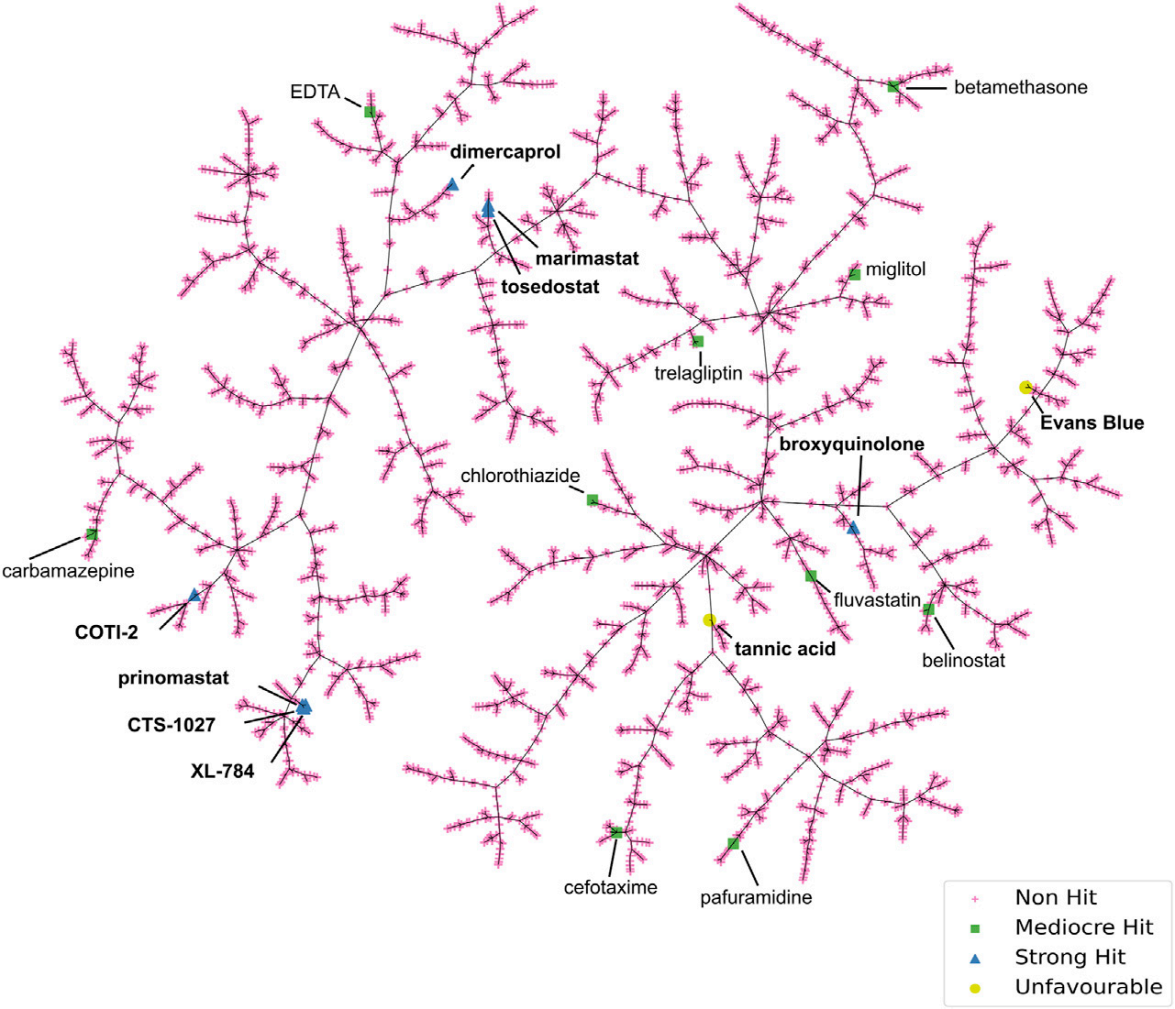
Tree Manifold Approximation Projection (TMAP) representation of the chemical space covered by the HTS library. Non-hits are shown as pink crosses, mediocre hits are shown as green squares, and strong hits are shown as blue triangles. Unfavourable hits are shown as yellow circles.

**FIGURE 7:**
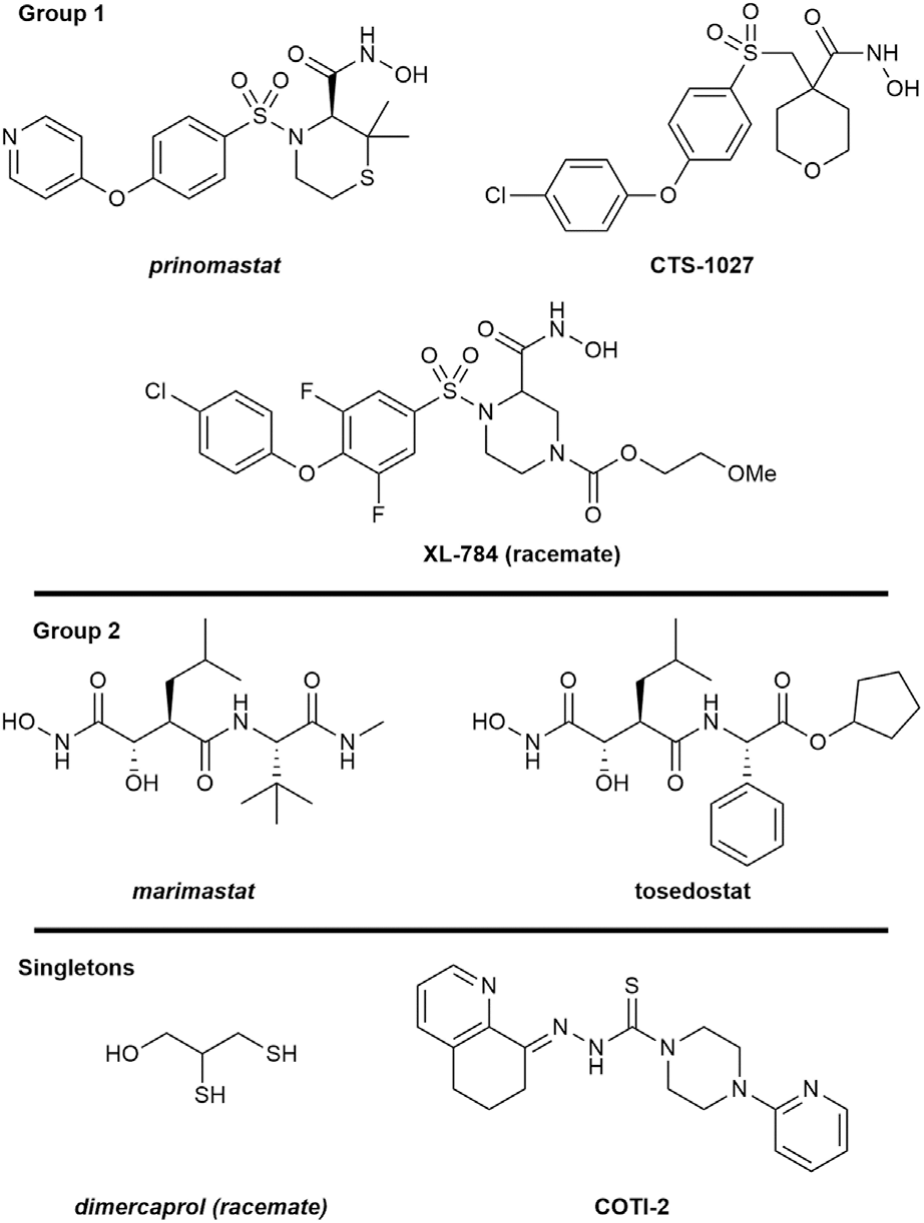
Structures of the seven lead SVMP compounds including three known inhibitors (italics) and four novel hits with nanomolar EC_50_s grouped by structural similarity determined by TMAP. Structures are drawn by ChemDraw based on SMILES provided by the source of the drug screening library.

To scrutinise this repurposed library from a structural activity relationship (SAR) perspective, we used Tree Manifold Approximation Projection (TMAP) to visualise the HTS chemical space explored and to identify trends among closely related scaffolds (Probst and Reymond, 2020). Briefly, TMAP displays chemical space as a tree manifold displaying similar groups of compounds as branches while the distance between each compound represents similarity–we would therefore expect similar compounds to exist within the same sub-branches. TMAP representation of our dataset showed that the majority of the mediocre hits detected were structurally distinct from other hits (Figure 6). However, for the lead compounds, which included the four novel hits with nanomolar EC_50_s (XL-784, CTS-1027, COTI-2 and tosedostat) and the known SVMP inhibitors (marimastat, prinomastat and dimercaprol), this approach allowed identification of two distinct groups: i) CTS-1027, XL-784 and prinomastat—all lipophilic non-peptidomimetic hydroxamate MMPis (Johnson et al., 2006; Kahraman et al., 2009) and ii) marimastat and tosedostat—an established peptidomimetic SVMP inhibitor and a structurally similar compound, respectively. Dimercaprol and COTI-2 are visibly isolated from the other hits in separate branches and exist as singletons. This reduces confidence in the latter compounds as starting points for optimisation due to lack of evidence of an SVMP-inhibitory scaffold, with their closest neighbours in the HTS showing no SVMP-inhibitory activity. This hypothesis is further supported by the poorer performance of COTI-2 and dimercaprol in secondary EC_50_ screens compared to other hits. The structures of the lead compounds are presented in Figure 7.

### 3.5 Assessment of absorption, distribution, metabolism and elimination (ADME) properties of the hit compounds

**FIGURE 8:**
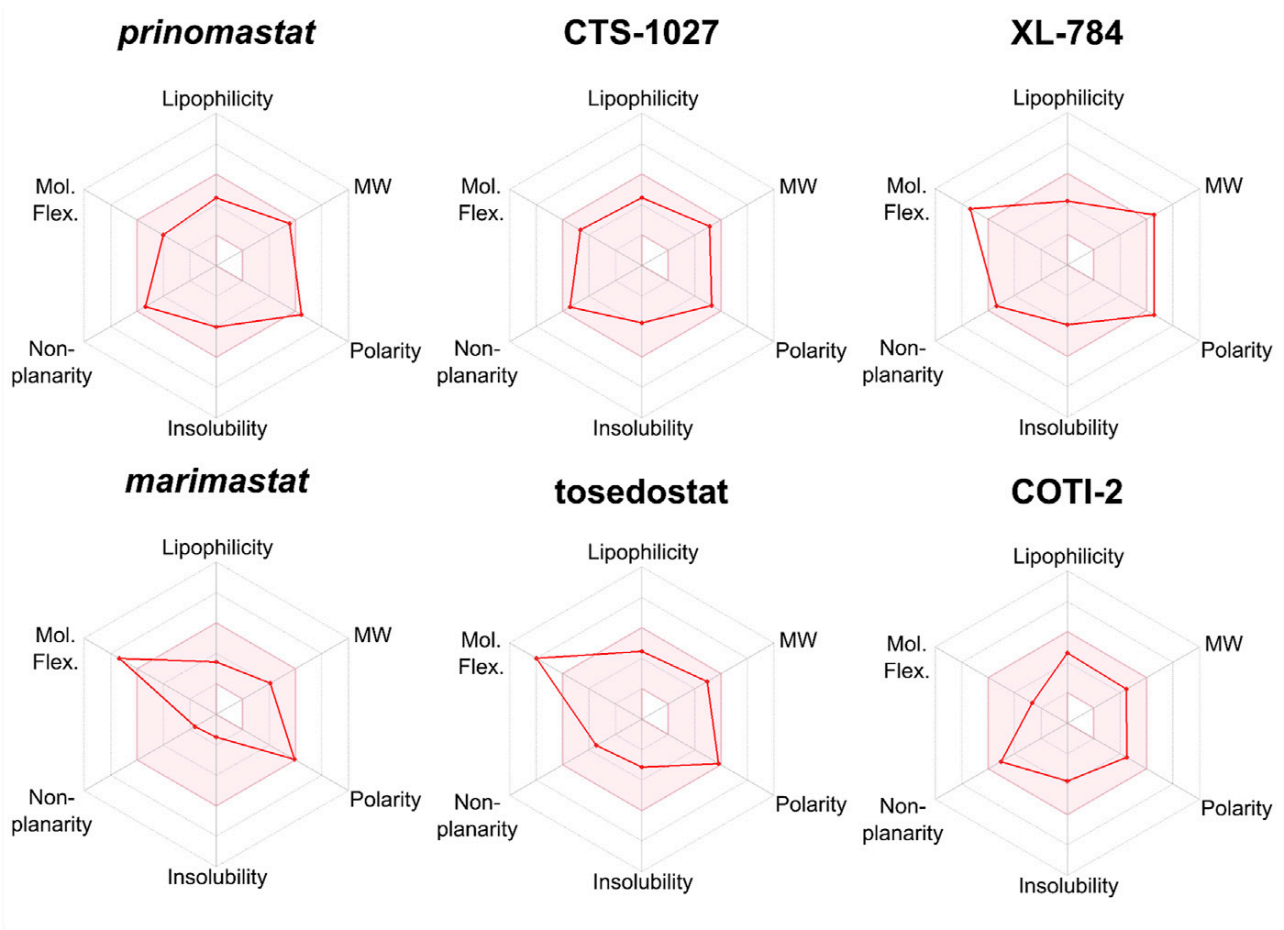
Radar plots generated by SwissADME for lead compounds of interest. The shaded area represents the ideal range for oral bioavailability. Thresholds: Lipophilicity: XLOGP3 between −0.7 and 5.0, Molecular Weight (MW): 150 < MW < 500 g/mol; Polarity: 20 < TPSA 130 Å^2^, Insolubility: −log *S* < 6, non-planarity: fraction of carbons in the sp3 hybridization not less than 0.25, and Molecular Flexibility (Mol.Flex.): no more than 9 rotatable bonds.

We used SwissADME to evaluate the predicted physicochemical properties and drug-likeness of the seven lead compounds (Figure 7) using their validated machine-learning models (Daina et al., 2017). Predicted values indicating lipophilicity (XLOGP3), molecular weight (MW), polarity (topological polar surface area, TPSA), non-planarity (fraction of carbons in the sp3 hybridisation state, Fsp3), molecular flexibility (number of rotatable bonds, RotB) and solubility are provided in Table 2, alongside radar plots for visualisation in Figure 8.

**TABLE 2:** Selected SwissADME predicted properties of the seven lead SVMP compounds including three known inhibitors (italics) and four novel inhibitors. Properties of concern based on violations of drug-likeness rules are indicated in bold.

Drug	Lipophilicity	MW	Polarity	Non-planarity	Molecular flexibility	Drug likeness rule violations				Solubility
						Lipinski	Ghose	Veber	Egan	
*Prinomastat*	2.26	460.0	**142.5**	0.33	6	0	0	1	1	Moderate
CTS-1027	2.27	425.9	110.3	0.32	7	0	0	0	0	Soluble
XL-784	1.85	**548.9**	**145.9**	0.33	**11**	2	1	2	1	Soluble
*Marimastat*	0.51	331.4	127.8	0.80	**11**	0	0	1	0	Very soluble
Tosedostat	2.29	406.5	125.0	0.57	**12**	0	0	1	0	Soluble
*Dimercaprol*	0.19	**124.2**	97.8	1.00	2	0	3	0	0	Very soluble
COTI-2	2.54	366.5	88.7	0.37	4	0	0	0	0	Soluble

Lipophilicity: XLOGP3 is a method of LogP calculation based on the number of heteroatoms and number of carbon atoms. MW: molecular weight. Polarity: measured as the topological polar surface area (TPSA). Non-planarity: the fraction of carbons with sp3 hybridisation. Molecular flexibility: the number of rotatable bonds. Drug-likeness rule violations: the values represent the number of rule violations. Lipinski’s rule: MW ≤ 500 g/mol, LogP ≤ 4.15, hydrogen bond acceptors ≤ 10 and hydrogen bond donors ≤ 5. Ghose filter: 160 ≤ MW ≤ 480, LogP ≤ 5.6, and 40 ≤ molar refractivity ≤ 130 and 20 ≤ number of atoms ≤ 70. Veber’s rule: RotB ≤ 10 and TPSA ≤ 140. Egan’s filter LogP≤ 5.88 and TPSA ≤ 131.6. Solubility: calculated using the widely used ESOL, dataset and model. log S scale: insoluble (<−10), poorly soluble (−10 to −6), moderately soluble (−6 to −4), soluble (−4 to −2), very soluble -(-2 to 0) and highly soluble (>0).

SwissADME assesses drug-likeness through design rules as determined by Lipinski, Ghose, Veber, and Egan (Ghose et al., 1999; Egan et al., 2000; Lipinski et al., 2001; Veber et al., 2002). These rules use the predicted ADME properties and defined thresholds to determine whether a compound violates a rule. The number of violations of these rules is summarised in Table 2. All compounds possess no greater than 2 violations to any rule with the exception of dimercaprol which possesses three Ghose violations due to molecular weight < 160 Da, molar refractivity < 40 and total number of atoms < 20 (Ghose et al., 1999). CTS-1027 and COTI-2 have no violations across all 4 rules, whilst marimastat and tosedostat have a single violation in Veber’s rule due to having the number of rotatable bonds as > 9 (molecular flexibility). Prinomastat has one violation in both Veber and Ghose design rules due to its polarity (TPSA = 142.5 Å^2^), though considering the thresholds of these rules (TPSA > 140 Å^2^ and 131.6 Å^2^, respectively) these violations are minor. XL-784 is the only compound to have violations across all four rules. This is due to its high molecular weight and number of hydrogen bond donors (Lipinski and Ghose), as well as its polarity and flexibility (Veber and Egan).

Outside the parameters used to determine rule violations, SwissADME also profiles solubility by three methods; an implementation of the ESOL method (Delaney, 2004), a model adapted from (Ali et al., 2012), and a method developed by SILICOS-IT (Daina et al., 2017). Low solubility of molecules may cause issues with absorption. No hit compounds were found to be poorly soluble (Table 2) supporting the insight from design rules that the majority of hits are unlikely to experience issues with absorption. Overall, assessment of predicted ADME properties suggests that the novel hits CTS-1027, COTI-2 and tosedostat are the most promising hits for optimisation, while XL-784 would require more extensive optimisation of its ADME properties.

## 4 Discussion

SVMPs are a major snake venom toxin family responsible for causing substantial pathology in snakebite patients, while conventional antivenom therapies are often associated with a high incidence of adverse effects, as well as financial and logistical constraints. Consequently, efforts are being made to find alternative therapies that could overcome these existing barriers for victims of snakebite. Through validated miniaturisation of an existing fluorometric assay and the application of liquid handling instruments, we have developed a high-throughput screening platform with the capacity to screen ∼7,000 individual compounds against a venom of interest in a single day. Utilising this HTS platform, we screened 3,547 post-Phase I compounds in singleton at 10 µM against five medically important viper venoms of diverse taxonomy, geographic location and SVMP content. The robust nature of this HTS is quantified by consistently high Z′ across assay plates (0.71–0.96), providing confidence in the results obtained. Out of the 0.4% hit rate from the primary screen (14 strong pan-species inhibitors), after secondary testing and assessment of physiochemical properties, four discrete clusters of structurally similar strong (nM EC_50_s) hit compounds were identified (Figures 6, 7). Three hit compounds (XL-784, CTS-1027 and tosedostat) shared structural similarities with MMP inhibitors previously shown to also inhibit SVMPs (marimastat and prinomastat (Howes et al., 2007; Arias et al., 2017; Albulescu et al., 2020b; Menzies et al., 2022)). XL-784, first prepared by Exelixis, and CTS-1027 (also known as RS-130830), originally prepared by Roche Bio-Science, are both known MMP inhibitors, while both tosedostat and COTI-2 have unknown MMP inhibitory activity. Due to the less favourable EC_50_ performance in the secondary screen, COTI-2 was deprioritised as a backup compound.

Despite promising inhibitory potencies and ADME properties, tosedostat retains a major limitation as a future SVMP inhibiting drug. Tosedostat is a pro-drug that is metabolised into its active form, which is an aminopeptidase inhibitor investigated for cancer indications. This transformation involves cleavage of a labile ester linkage by serum esterases to reveal a carboxylic acid necessary for its aminopeptidase inhibitory function (Krige et al., 2008). As our HTS was not carried out under the appropriate metabolic conditions to generate tosedostat’s active metabolite, it can be assumed that the anti-SVMP activity of this drug is mediated by its ester form. For our purposes, hydrolysis to the free acid would add unnecessary polar functionality which may have negative implications on absorption, metabolism, and overall exposure at the target SVMPs *in vivo*. For these reasons, we have deprioritised tosedostat for onward development.

The ADME properties described in Table 2 and Figures 7, 8 suggest that XL-784 is perhaps less attractive due to its high molecular weight, polarity, molecular flexibility, and number of hydrogen bond acceptors—all of which are associated with poor bioavailability, and XL-784 also has a reported low aqueous solubility of 20 μg/mL (Williams et al., 2011b). For these reasons, analogues of XL-784 with improved pharmacokinetic properties are being evaluated in our medicinal chemistry program. CTS-1027 and prinomastat provide the best medicinal chemistry starting points for future SVMP inhibitor discovery due to their desirable physicochemical and structural properties and minor violations of drug-likeness rules. However, it should be noted that CTS-1027 and prinomastat were flagged in SwissADME as having toxicity and pharmacokinetic (PK) concerns. Prinomastat previously progressed into Phase III trials where musculoskeletal toxicity was observed in a significant proportion of patients that received a treatment course of 15 mg bi-daily dosing for 3–36 months, as well as an increased risk of venous thromboembolic disease in cancer patients receiving combination chemotherapy (Bissett et al., 2005). It is anticipated that these side effects were due to inhibition of MMP-1. Consequently, a more selective MMP/SVMP inhibitor will be pursued in our drug discovery campaign to avoid joint toxicities. CTS-1027 previously entered Phase II clinical trials for the treatment of hepatitis C, and a range of serious adverse events were associated with the administration of 30 mg per day for up to 24 weeks (e.g., single incidences of anaemia, hematemesis, upper GI haemorrhage, chest pain, staphylococcal abscess, prostate cancer and chronic obstructive pulmonary disease) (Conatus Pharmaceuticals Inc, 2012). It should be noted that these toxicity concerns were associated with a long-term treatment course and high drug dosage, whereas projected snakebite therapy would likely only involve short course dosing for perhaps up to 72 h. Additionally, these side effects are reversible following treatment termination, thereby avoiding permanent pathologies (Scatena, 2000).

The HTS assay described here identifies compounds that directly inhibit the enzymatic activity of SVMP toxins (Neumann et al., 2004; Gutiérrez et al., 2021). One limitation of this assay is that compounds that inhibit SVMP activity through metal chelation will not be observed as highly potent in our HTS and thus may not be selected as hits for progression. This is important to note as the metal chelator dimercaprol has been shown to successfully inhibit SVMP activity *in vivo,* with the derivative DMPS currently being planned for progression into Phase II clinical trials for snakebite (Abouyannis et al., 2022). Therefore, screening to identify compounds with similar mode of action would require an alternative screening model that better represents the phenotypic nature of snakebite envenoming. Another caveat to consider is that fluorescence-based drug screens are vulnerable to interference from compounds that are either fluorescent quenchers (false positives) or auto-fluorescent (false negatives) (Yang et al., 2020). Diversity based screening libraries often contain compounds with several aromatic rings in their structure which are inherently fluorescent in the lower-blue range of the spectrum (excitation ∼ 350 nm and emission of 450–495 nm) (Thorne et al., 2010). The emission wavelength of this assay is 410–420 nm, therefore such false negatives are likely to be minimal. Chemical scrutiny of hits prior to chemical selection can be used to identify quenching moieties and deprioritised for onward progression, while false negatives with auto-fluorescent characteristics could be progressed into secondary assays if they possessed promising structural similarities to known inhibitors or demonstrate inhibitory activity in orthogonal, non-fluorescent assays.

Prior to the initiation of medicinal chemistry campaigns around the two hit-to-lead compounds described here (CTS-1027 and prinomastat), traction along a defined drug discovery pipeline would be essential, inclusive of activity confirmation in additional *in vitro* assays and critical evaluation PK/PD properties prior to progression into *in vivo* testing. Although we do not anticipate dropouts in *in vivo* experiments (MMPis such as marimastat and metal chelators such as DMPS have previously displayed activity in murine envenoming models (Albulescu et al., 2020a; 2020b; Menzies et al., 2022)), discrepancies between *in vitro* enzymatic screening and results from *in vivo* testing could occur due to the increased degree of complexity of venom pathology in a biological system rather than an enzymatic *in vitro* setting. For example, SVMPs directly target the coagulation cascade through the activation of factor X and/or prothrombin, which are effects that this current enzymatic HTS platform cannot determine. Marimastat and prinomastat are known to inhibit SVMP activation of the clotting cascade, so a further set of experiments to confirm that hits identified in this assay show similar inhibition of coagulopathy would be beneficial as a tertiary screen (Mackessy and Stephen, 2009; Cabral-Pacheco et al., 2020). In addition to tertiary *in vitro* testing, the pharmacokinetic and pharmacodynamic (PK/PD) properties of the lead compounds need to be considered to allow for rational dose design prior to progression into preclinical *in vivo* models. One benefit of screening repurposed compound libraries, such as in this HTS study, is that many of the compounds involved will have already been subjected to human safety and efficacy testing, which will provide data of value for considering human dose prediction.

As described above, we have identified two novel lead candidates which are poised for progression along our drug discovery pipeline (tertiary screening and PK/PD evaluation inclusive of informed dose selection). Prinomastat and CTS-1027 are viable as parent compounds for the establishment of medicinal chemistry campaigns to develop the first designer toxin-specific small molecule for the treatment of snakebite. To our knowledge, this is the first time a validated, scalable HTS platform has been demonstrated for the investigation of small molecule inhibitors against medically relevant snake venom toxins. We hope that this work will help to overcome barriers of throughput and accelerate the future identification of alternative snakebite therapeutics. In addition to small molecules, this platform is amenable to use to discover monoclonal antibody-based therapeutics (Knudsen and Laustsen, 2018; Laustsen, 2018; Harrison and Gutiérrez, 2019; Knudsen et al., 2019), as well as for high-throughput profiling of SVMP activity from a diversity of venoms (e.g., macroevolutionary or population-level analyses). The platform can also be utilised for libraries of any size; smaller, rationally selected libraries can be screened rapidly (e.g., analogues from a medicinal chemistry campaign) and progressed directly to secondary EC_50_ screening, whilst the scalability of this platform makes it equally applicable to larger, more diverse libraries such as the ReFRAME collection (12,000 small molecules which have reached clinical development or undergone significant preclinical development (Janes et al., 2018)) and AstraZeneca’s open innovation programme (AstraZeneca, 2022). Furthermore, it is feasible this HTS could be scaled up further based on the precedence for ultra-HTS discovery programmes which use the same fluorescent substrate (Madoux et al., 2016; Hou et al., 2021).

Our ongoing work aims to develop and validate a drug discovery pipeline from primary *in vitro* HTS through to preclinical testing, paving the way for the discovery and development of novel snakebite therapeutics against pathogenically important snake venom toxins. The automated set up of this HTS platform has also been applied in parallel to other toxin families, such as the PLA_2_s (Albulescu et al., 2020b). Collectively, these studies have the potential to vastly broaden the portfolio of preclinical stage small molecules for snakebite with options for novel drug combinations directed at distinct snake venom components. Following the long-term goal of successful progression through snakebite targeted clinical development regulatory approved oral drugs that can be rapidly delivered in the community soon after a bite to alleviate the severe, often life-threatening effects would provide a paradigm shift in snakebite envenoming therapeutics (Clare et al., 2021; Gutiérrez et al., 2021; Abouyannis et al., 2022; Carter et al., 2022).

## Data Availability

The original contributions presented in the study are included in the article/Supplementary Material, further inquiries can be directed to the corresponding author.

## Supplementary material

SUPPLEMENTARY FIGURE S1 Full-plate control data separated by column and row to demonstrate inter- and intra-plate consistency. **(A)** Graphs demonstrating the raw data from three independently-ran control plates, containing either full maximum signal (vehicle: DMSO) or full baseline signal (10 µM marimastat), with values split by row (left) or column (right). Consistently high vehicle values and consistently baseline marimastat values can be seen irrespective of where on the plate they are located. **(B)** Normalised data from the same experiments as shown in **(A)**. Normalisation of this data reduces inter-plate variation, as can be seen with plate 1 compared to plates 2 and 3 in **(A)**, without affecting the separation of the positive and negative control signal windows.

SUPPLEMENTARY FIGURE S2 Interleaved control data separated by column and row to demonstrate inter- and intra-plate consistency. Graphs demonstrating the normalised results from three independently-ran control plates, containing alternating rows of maximum signal (vehicle: DMSO), baseline signal (10 µM marimastat), venom vehicle (PBS) or a suboptimal dose of marimastat (50 nM). Values ordered by row (top) or column (bottom). As with Supplementary Figure S1, consistency can be seen in matched conditions regardless of the location of the well on the plate, as well as between independently repeated plates. The suboptimal dose of marimastat selected demonstrates that it inhibits at around 50% that of the baseline concentration.

SUPPLEMENTARY DATASHEET S1 Repurposed Drug library—The details provided by MedChemExpress of the bespoke 3,547 repurposed drug library created for snakebite drug discovery screening are displayed. This is inclusive of chemical structural data through simplified molecular-input line-entry system (SMILES) keys (column K) and CAS number.
