# Supplementary figures and images for: Snakebite drug discovery: high-throughput screening to identify novel snake venom metalloproteinase toxin inhibitors
Source: Front Pharmacol. 2024 Jan 11;14:1328950. doi: 10.3389/fphar.2023.1328950 (PMC10808794; doi:10.3389/fphar.2023.1328950)

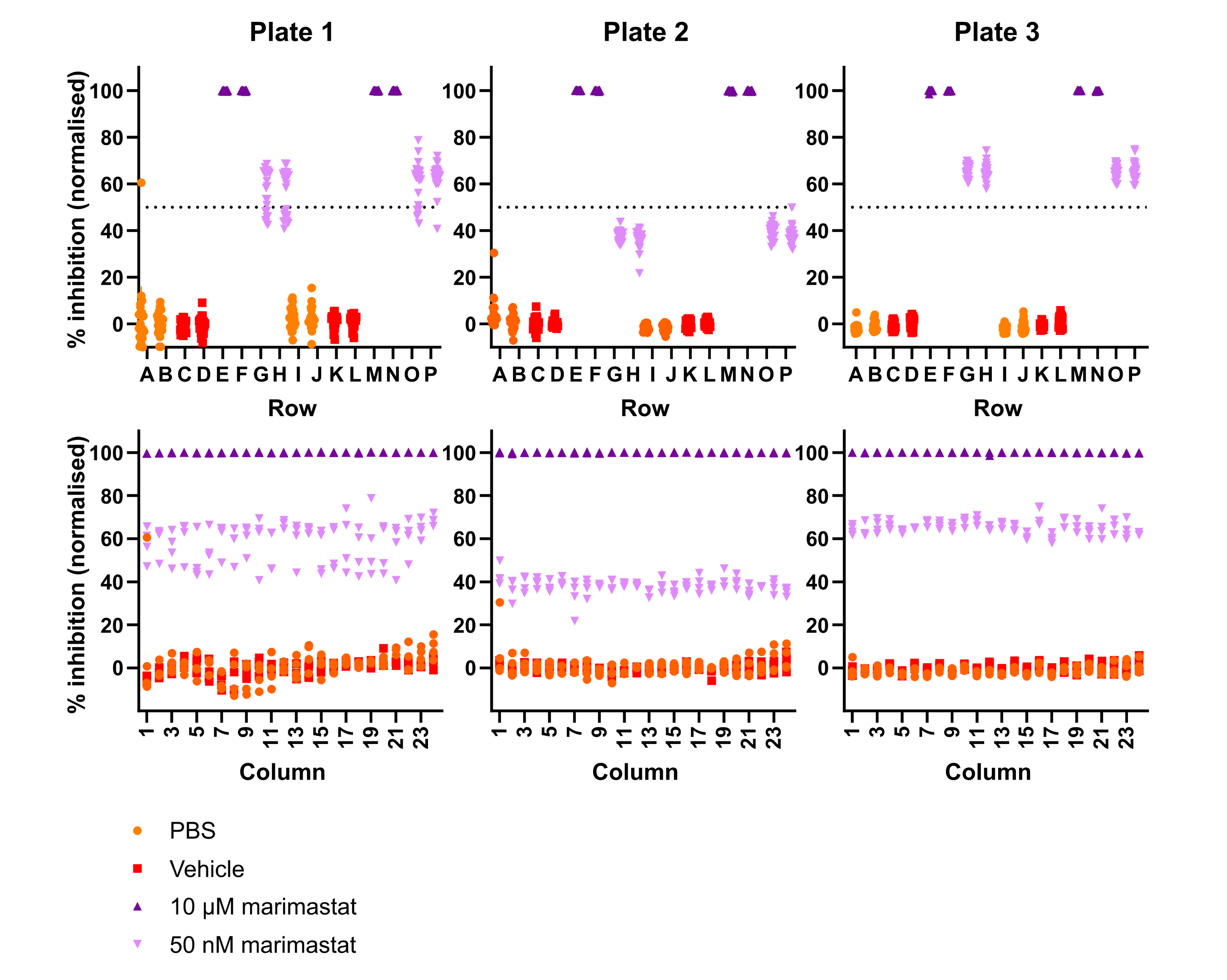

Supplement: Supplementary file 1 [file Image2.tif]

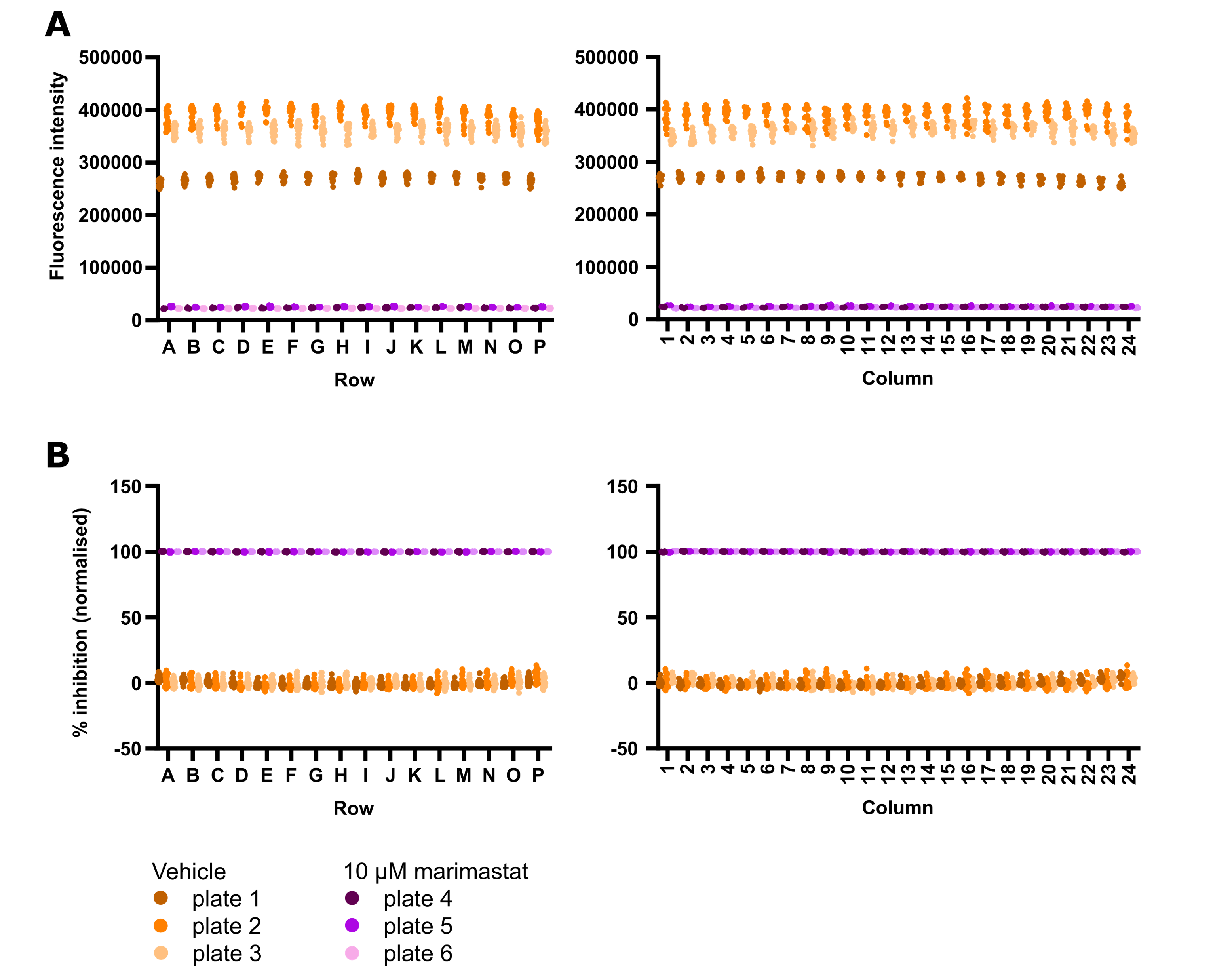

Supplement: Supplementary file 2 [file Image1.tif]
